# The PKR activator, PACT, becomes a PKR inhibitor during HIV-1 replication

**DOI:** 10.1186/1742-4690-10-96

**Published:** 2013-09-11

**Authors:** Guerline Clerzius, Eileen Shaw, Aïcha Daher, Samantha Burugu, Jean-François Gélinas, Thornin Ear, Lucile Sinck, Jean-Pierre Routy, Andrew J Mouland, Rekha C Patel, Anne Gatignol

**Affiliations:** 1 Lady Davis Institute for Medical Research, 3999 Côte Ste Catherine, Montréal, QC H3T 1E2, Canada; 2 Department of Medicine, Division of Experimental Medicine McGill University, Montréal, Québec, Canada; 3 Depatment of Microbiology & Immunology, McGill University, Montréal, Québec, Canada; 4 Chronic viral Illness Service and Division of Hematology, McGill University Health Centre, Montréal, Québec, Canada; 5 Department of Biological Sciences, University of South Carolina, Columbia, SC, USA; 6 Present address: Radiation Oncology Department, Jewish General Hospital, Montréal, Canada; 7 Present address: Gene Medicine research group, John Radcliffe Hospital, University of Oxford, Oxford, UK

**Keywords:** Human immunodeficiency virus type 1 (HIV-1), Protein kinase RNA-activated (PKR), PKR activator (PACT), Eukaryotic translation initiation factor 2 (eIF2α), Lymphocytes, Astrocytes

## Abstract

**Background:**

HIV-1 translation is modulated by the activation of the interferon (IFN)-inducible Protein Kinase RNA-activated (PKR). PKR phosphorylates its downstream targets, including the alpha subunit of the eukaryotic translation Initiation Factor 2 (eIF2α), which decreases viral replication. The PKR Activator (PACT) is known to activate PKR after a cellular stress. In lymphocytic cell lines, HIV-1 activates PKR only transiently and not when cells replicate the virus at high levels. The regulation of this activation is due to a combination of viral and cellular factors that have been only partially identified.

**Results:**

PKR is transiently induced and activated in peripheral blood mononuclear cells after HIV-1 infection. The addition of IFN reduces viral replication, and induces both the production and phosphorylation of PKR. In lymphocytic Jurkat cells infected by HIV-1, a multiprotein complex around PKR contains the double-stranded RNA binding proteins (dsRBPs), adenosine deaminase acting on RNA (ADAR)1 and PACT. In HEK 293T cells transfected with an HIV-1 molecular clone, PACT unexpectedly inhibited PKR and eIF2α phosphorylation and increased HIV-1 protein expression and virion production in the presence of either endogenous PKR alone or overexpressed PKR. The comparison between different dsRBPs showed that ADAR1, TAR RNA Binding Protein (TRBP) and PACT inhibit PKR and eIF2α phosphorylation in HIV-infected cells, whereas Staufen1 did not. Individual or a combination of short hairpin RNAs against PACT or ADAR1 decreased HIV-1 protein expression. In the astrocytic cell line U251MG, which weakly expresses TRBP, PACT mediated an increased HIV-1 protein expression and a decreased PKR phosphorylation. In these cells, a truncated PACT, which constitutively activates PKR in non-infected cells showed no activity on either PKR or HIV-1 protein expression. Finally, PACT and ADAR1 interact with each other in the absence of RNAs.

**Conclusion:**

In contrast to its previously described activity, PACT contributes to PKR dephosphorylation during HIV-1 replication. This activity is in addition to its heterodimer formation with TRBP and could be due to its binding to ADAR1. HIV-1 has evolved to replicate in cells with high levels of TRBP, to induce the expression of ADAR1 and to change the function of PACT for PKR inhibition and increased replication.

## Background

Human immunodeficiency virus type 1 (HIV-1) mRNA expression is controlled at the transcriptional, processing and translational levels [1-3]. The main translational mechanism is a cap-mediated scanning from its 5’ end but additional mechanisms occur including internal ribosome entry site in gag, programmed −1 ribosomal frameshift to produce Gag-Pol and discontinuous ribosome scanning to translate Env [4-6]. HIV-1 translation is modulated by viral components, like Trans-Activation Response element (TAR) RNA [7-10] and by cellular factors including translation factors, Protein Kinase RNA activated (PKR), TAR RNA Binding Protein (TRBP), PKR Activator (PACT), the La autoantigen, Staufen1 and the Adenosine Deaminase Acting on RNA (ADAR)1 [9,11-15]. The positive factors act by releasing the block due to the TAR structure, by inhibiting PKR or by inhibiting PACT [7-9,16-20].

The interferon (IFN)-inducible PKR is a key double-stranded RNA-binding protein (dsRBP), and a serine/threonine kinase. Its activation leads to autophosphorylation and the phosphorylation of its downstream targets, including the alpha subunit of the eukaryotic translation initiation factor 2 (eIF2α). Phosphorylated eIF2α (P-eIF2α) prevents translational initiation of viral and cellular mRNAs. PKR is central in the host innate defense strategies with strong antiviral and antigrowth activities [21-24]. In addition, its N-terminus forms a complex with proteins involved in cellular signaling pathways to mediate the activation of the NF-κB protein complex, which contributes to the induction of inflammatory cytokines [25,26]. PKR is extremely effective in restricting HIV-1 expression and replication *in vitro*[12,19,27-30]. Despite this observed activity, HIV-1 replicates efficiently in many permissive cell lines and primary cells, suggesting that the kinase activity of PKR in natural infection of lymphocytes is tightly regulated [17].

Many viruses that replicate efficiently have means to inactivate PKR and the HIV-1 Tat protein is one of these countermeasures [31-33]. Cells also avoid PKR activation using dsRNA sequestration or protein-protein interactions, likely as a normal process to allow their growth. Examples of direct interaction include the cellular protein p58^IPK^, which binds to PKR and prevents its dimerization, tRNA-dihydrouridine synthetase-2, TRBP and ADAR1, which bind through their dsRNA Binding Domains (dsRBDs) and exert a strong inhibitory activity [12,19,22,32,34-40]. Besides dsRNA and heparin, PACT, the cytokine MDA-7/interleukin 24 and the transcription factor E2F-1 induce PKR activation [32,41-45]. PKR activation upon virus infection is also observed in some specialized cells. For example, cardiomyocytes are cells with high activation of PKR and PKR-like ER protein kinase (PERK) upon coxsackievirus infection due to a downregulation of p58^IPK^ by the virus [46]. Similarly, astrocytic cells represent an example of naturally HIV-resistant cells with high PKR activation. In these cells, TRBP is expressed at very low amounts and cannot counteract PKR activation induced by the virus [47-49]. In contrast, in HIV-infected lymphocytes PKR activation is reduced when the virus reaches high concentrations and this is due in parts to the expression of TRBP, ADAR2 and to an increased ADAR1 expression that inhibits PKR activation [12,17,50,51].

PACT and its murine homolog RAX, are stress-inducible PKR activators [42,44,52]. They are proapoptotic proteins that induce apoptosis upon cellular stress by PKR activation [52-54]. PACT has two dsRBDs and a C-terminus domain called Medipal by homology with TRBP. All three domains in PACT homodimerize and interact with PKR and TRBP [20,55,56]. The Medipal domain mediates activation of PKR or inhibition by TRBP [18,55,57-59]. A cellular stress dissociates TRBP-PACT interactions and allows PACT activation of PKR. Therefore, PACT acts as a PKR activator in cells with low TRBP concentration or after stress induction, whereas it acts as a PKR inhibitor in cells with high TRBP content [13,18,20,55,60]. Its activity has not been tested in HIV-infected cells. Here, we observed that PKR is transiently induced and activated in HIV-1 infected peripheral blood mononuclear cells (PBMCs) with increased expression of both ADAR1 and PACT. We show that PACT binds to PKR during HIV-1 infection and that its activity is changed from an activator into an inhibitor of PKR in HIV-permissive cells and in astrocytic cells, which do not replicate HIV-1 efficiently. This change of function may be related to an interaction between ADAR1 and PACT.

## Results

### PKR is transiently induced and activated in PBMCs at the beginning of HIV-1 infection

**Figure 1:**
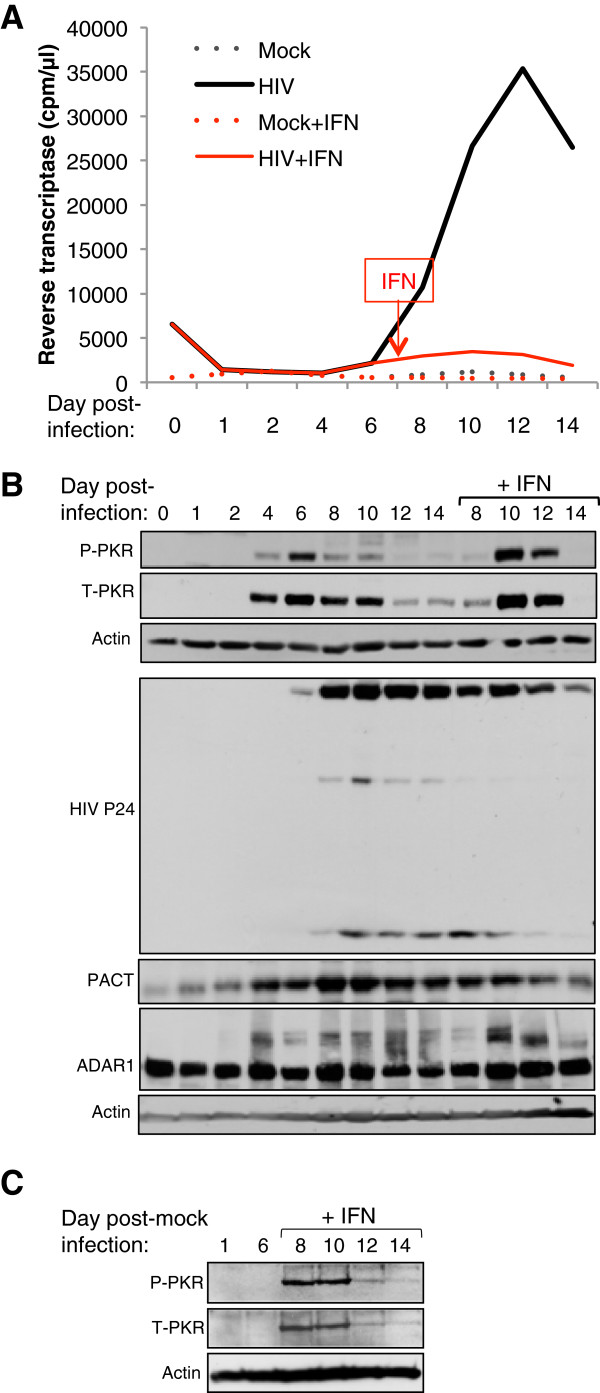
**PKR is activated after HIV-1 infection and inhibited during active HIV-1 replication. A)** HIV-1 pNL4-3 kinetics from infected PBMCs. 6.5 × 10^7^ PBMCs from a healthy donor were infected with HIV-1 pNL4-3. At day 7, cells were separated in two flasks and IFN α/β (10000U/mL) was added to the cells in one of them up to day 14. Aliquots of cell supernatant were collected at different times and assayed for RT activity. **B)** Protein expression of pNL4-3-infected PBMCs. 50 μg of whole-cell extracts from pNL4-3-infected PBMCs from different harvest times were subjected to a 10% SDS PAGE and blotted with anti-P-PKR, anti-PKR, anti-HIV-p24, anti-PACT, anti-ADAR1 and anti-actin antibodies as indicated. **C)** Protein expression of mock-infected PBMCs. 6.5 × 10^7^ PBMCs from the same donor as in B were cultured and passed at the same time as in B. IFN α/β was added similarly from day 7 to 14. 50 μg of whole-cell extracts from mock-infected PBMCs from the indicated times were subjected to a 10% SDS PAGE and blotted with anti-P-PKR, anti-PKR and anti-actin antibodies as indicated.

We have previously shown that HIV-1 infection of the lymphocytic Jurkat T cell line induces PKR activation during the first days of infection, followed by an inactivation during high HIV-1 replication [12]. To determine if this regulation is also true in primary cells, we infected PBMCs from healthy donors with the pNL4-3 HIV-1 clone (Figure 1). To determine the importance of PKR activation during an IFN response in these cells and its impact on HIV-1 replication, half of the culture was treated with IFN at day 7 and IFN was maintained in the medium up to day 14. Following viral infection, reverse transcriptase (RT) activity was visible at day 6 and reached a peak at day 12, whereas the addition of IFN at day 7 induced a dramatic decrease in RT activity at day 8 (Figure 1A). Cell samples were gathered every two days and analyzed by Western blotting (Figure 1B). We first observed a very low basal level of PKR in uninfected cells (D0). PKR expression was induced from day 4 to 10 with a higher induction at day 6. It was activated (P-PKR) mainly at day 6 followed by deactivation. In contrast, when IFN was added at day 7, PKR was induced and activated 3 days after (D10-12). Because ADAR1 expression is induced upon HIV-1 infection in Jurkat cells [12], we also evaluated its expression in this experiment. We found that ADAR1 p150 was induced at day 4 and was maintained up to day 14. IFN further induced its expression at day 10. To determine if PACT could likewise have a role in the regulation of PKR during HIV-1 infection of PBMCs, we also evaluated its expression. Surprisingly, we found an increase in PACT expression concomitant with ADAR1 p150 increase just before the expression of Gag protein was visible. In this case, PACT was not further induced by IFN. Interestingly, in a mock infection of the same cells, PKR was induced and activated one day after the addition of IFN (Figure 1C), suggesting that this induction is delayed by two days in HIV-1 infected cells. These results show that, PKR is transiently induced and activated in primary lymphocytes and deactivated when the virus replicates actively and that ADAR1 and PACT may play a role in this regulation.

### PACT belongs to a multiprotein complex formed around PKR during HIV-1 infection

**Figure 2:**
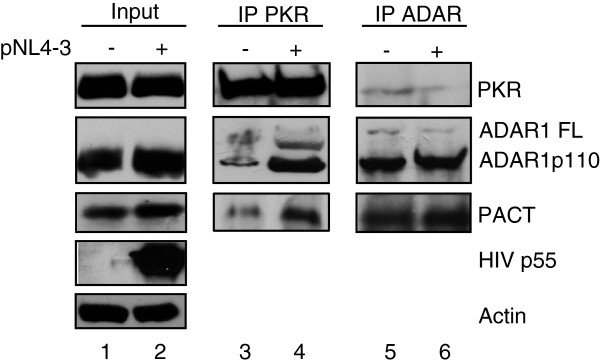
**Increased ADAR1-PKR and PACT-PKR interactions during HIV-1 infection.** Jurkat cells were mock-infected or infected with HIV-1 pNL4-3. Cell lysates were collected at day 15, which corresponds to the peak of infection for the pNL4-3-infected Jurkat cells. These were immunoprecipitated with anti-PKR or anti-ADAR1. 50 μg of proteins from each lysate (input; lanes 1–2) and the PKR (lanes 3–4) or ADAR (lanes 5–6) immunoprecipitated complexes were run on a 10% SDS-PAGE and blotted using anti-PKR, anti-ADAR1 (against full-length p150 and p110), anti-PACT, anti-HIV-p24 and anti-actin.

Many viral and cellular factors prevent PKR activation resulting in active viral infections and cell growth [32,33]. In the case of HIV-1 infection, the viral protein Tat, large amounts of TAR RNA, cellular proteins TRBP and ADAR1 all contribute to PKR inhibition [17]. Because cells also express PKR activators, and because we observed an increase in PACT expression during HIV-1 infection, we questioned whether PACT could contribute to PKR activation to enhance cell response and balance its inhibition by other factors. We have previously demonstrated that PACT is an activator or an inhibitor of PKR depending on TRBP expression in stressed or non-stressed cells [18,20,55]. We also observed that PACT expression is slightly increased at the peak of infection in Jurkat cells (Figure 2, input). We next determined if PACT was present in the complex formed around PKR during HIV-1 infection of lymphocytes. By immunoprecipitation (IP) with a PKR antibody, we observed that PACT interaction with PKR is increased at the peak of infection (Figure 2). This result resembles the previously observed increase in ADAR1 production and interaction [12]. By IP with an ADAR1 antibody, we also found that PACT is in the same complex as ADAR1, therefore suggesting that these two proteins are part of the multiprotein complex around PKR in HIV-1 infected cells.

### PACT is a PKR inhibitor in HIV-1 transfected HEK 293T cells

**Figure 3:**
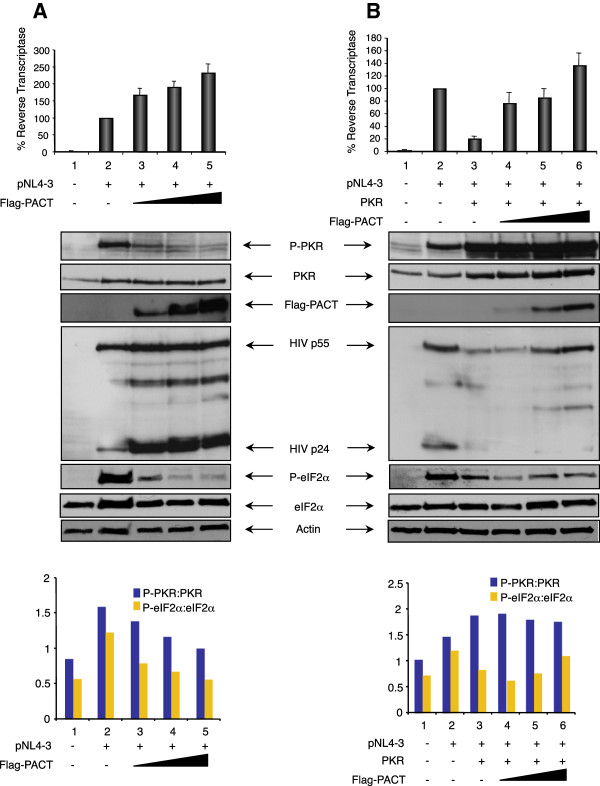
**PACT increases HIV-1 protein expression and virion production in HEK 293T cells by PKR and eIF2α inhibition. A)** PACT inhibits endogenous PKR and eIF2α phosphorylation and increases pNL4-3 expression. HEK 293 T cells were mock transfected (lane 1), transfected with 2 μg pNL4-3 (lanes 2–5) and 0.5 μg (lane 3), 1.0 μg (lane 4) or 1.5 μg (lane 5) of pCMV2-Flag-PACT. pCMV2 was added to reach the same amount of transfected DNA. (Top) % RT activity is the ratio between the RT level in the presence of PKR and PACT versus pNL4-3 alone normalized to 100%. Shown is the average of 6 independent transfections ± SEM. (Middle) Immunoblot of cell extracts of a representative experiment from the same transfected cells using antibodies against P-PKR, PKR, Flag, HIV-1 p24, P-eIF2α, eIF2α and actin. (Bottom) Ratio of phosphorylated PKR (P-PKR) versus PKR and P-eIF2α versus eIF2α. The band intensity was digitalized using Adobe Photoshop software from the bands shown above. P-PKR/PKR and P-eIF2α/eIF2α ratio was calculated by dividing the P-PKR or P-eIF2α intensity by the total PKR or eIF2α intensity of each band. **B)** PACT reverses PKR inhibition of pNL4-3. HEK 293 T cells were mock transfected (lane 1), transfected with 2 μg pNL4-3 (lanes 2–6), 0.5 μg pcDNA1-PKR (lanes 3–6), 0.5 μg (lane 4), 1.0 μg (lane 5) or 1.5 μg (lane 6) of pCMV2-Flag-PACT. Empty corresponding plasmids were added to reach the same amount of transfected DNA. (Top) % RT activity is calculated as in **A)**. Shown is the average of 6 independent transfections ± SEM. (Middle) Immunoblot of cell extracts of a representative experiment from the same transfected cells using the same antibodies as in **A)**. (Bottom) Ratio of phosphorylated PKR versus PKR and P-eIF2α versus eIF2α. The band intensities were calculated as in **A)**.

We next questioned whether the role of PACT in a complex with PKR during HIV-1 infection would be as an activator or an inhibitor. To determine this role on viral protein expression and virion production, we transfected HEK 293T cells with pNL4-3 in the absence or presence of transfected PKR and evaluated the activity of a PACT expressing vector on viral expression and on PKR activation (Figure 3). Transfection of the HIV-1 molecular clone induced PKR and eIF2α phosphorylation (Figure 3A and B, lane 2). When cells were transfected with pNL4-3 and PACT in the absence of overexpressed PKR, PACT was able to increase HIV-1 protein expression and virion production up to 2.3 fold (Figure 3A). Surprisingly, increasing amounts of PACT clearly prevented PKR and eIF2α phosphorylation, indicating that the protein acts as an inhibitor of endogenous PKR and contributes to the enhancement of HIV-1 translation and consequently to the increased virion production.

As previously observed [12,49], transfected PKR reduced the expression of HIV-1 proteins and viral production and we show here that this is due to the concomitant increase in the ratio between P-PKR and PKR (Figure 3B, lane 3). In this case, increasing amounts of PACT restored viral protein expression and virion production up to 7 fold over the PKR-inhibited RT amount. The large amount of PKR did not allow appropriate quantification of the P-PKR/PKR ratio, but the P-eIF2α/eIF2α ratio clearly indicated that low amounts of PACT prevented the phosphorylation of eIF2α and increasing amounts restored HIV-1 protein expression and virion production (Figure 3B).

### PACT, ADAR1 and TRBP inhibit PKR and eIF2α phosphorylation and increase HIV-1 protein expression

**Figure 4:**
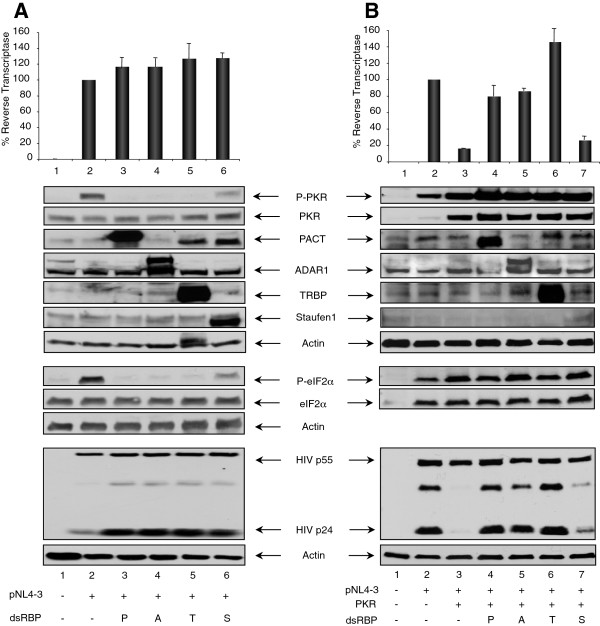
**PACT, ADAR1 and TRBP inhibit PKR activation in HIV-1 producing cells. A)** PACT, ADAR1 and TRBP, but not Staufen1, inhibit PKR and eIF2α phosphorylation. HEK 293T cells were mock transfected (lane 1), transfected with 2 μg pNL4-3 (lanes 2–6), and 1.5 μg of pCMV2-Flag-PACT (lane 3), pcDNA3.1-ADARp150-V5 (lane 4), pcDNA3-TRBP2 (lane 5) or pCMV-RSV-Staufen1-HA (lane 6). pcDNA3 was added to reach the same amount of transfected DNA. (Top) RT activity from cell supernatants normalized to 100% in the absence of PKR or dsRBPs. Shown is the average of 6 independent transfections ± SEM. (Bottom) Immunoblot of 150 μg of cell extract of a representative experiment from the same transfected cells using antibodies against P-PKR, PKR, P-eIF2α, eIF2α, PACT, ADAR1, TRBP, Staufen1, HIV-1 p24 and actin as indicated. Probing for P-eIF2α and eIF2α were performed on a separate membrane and the corresponding actin is shown. **B)** PACT, ADAR1 and TRBP, but not Staufen1, restore PKR inhibition of HIV-1 protein expression and virion production. HEK 293T cells were mock transfected (lane 1), transfected with 2 μg pNL4-3 (lane 2–7), 0.5 μg pcDNA1-PKR (lanes 3–7) and 1.5 μg of pCMV2-Flag-PACT (lane 4), pcDNA3.1-ADARp150-V5 (lane 5), pcDNA3-TRBP2 (lane 6) or pCMV-RSV-Staufen1-HA (lane 7). The empty pCMV2 vector was used to supplement transfections such that the same amount of DNA was transfected into each well. (Top) % RT activity is calculated as in **A)**. Shown is the average of 6 independent transfections ± SEM. (Bottom) 150 μg of each cell extract was analyzed by immunoblot against P-PKR, PKR, P-eIF2α, eIF2α, PACT, ADAR1, TRBP, Staufen1, HIV-1 p24 and actin as indicated. Shown is a representative experiment from the same transfected cells. Probing for P-eIF2α and eIF2α were performed on the same membrane as HIV-1 p24 and have the same actin.

To compare the activity of the different dsRBPs that contribute to HIV-1 expression and may inhibit PKR activation in HIV-1-infected cells, we next overexpressed PACT, ADAR1, TRBP and Staufen1 with pNL4-3 in the absence or presence of transfected PKR (Figure 4). When PKR was not overexpressed, all four proteins induced a mild increase of HIV-1 protein expression and virion production reflected by HIV-1 p24 expression in cells and RT assay in the supernatant (Figure 4A). In this assay, a dramatic difference was observed in PKR and eIF2α phosphorylation between the four dsRBPs. PACT, ADAR1 and TRBP completely inhibited PKR and eIF2α phosphorylation, whereas Staufen1 only induced a modest reduction, suggesting that the first three dsRBPs increase virus expression mainly by acting on PKR, whereas Staufen1 increases virus production by a PKR-independent mechanism. When PKR was overexpressed, PACT, ADAR1 and TRBP restored PKR-inhibited HIV-1 expression and virion production, but Staufen1 did not (Figure 4B). The level of HIV-1 p24 expression reflected a complete restoration of viral protein expression with PACT, ADAR1 and TRBP, but only a low increase by Staufen1 over PKR inhibition. The P-PKR/PKR and the P-eIF2α/eIF2α ratio were difficult to evaluate due to the high expression of transfected PKR, but suggests that PACT, ADAR1 and TRBP induce an additional mechanism, which also contributes to the restoration of viral expression in the context of overexpressed PKR.

### shRNAs against PACT and ADAR1 inhibit HIV-1 expression

**Figure 5:**
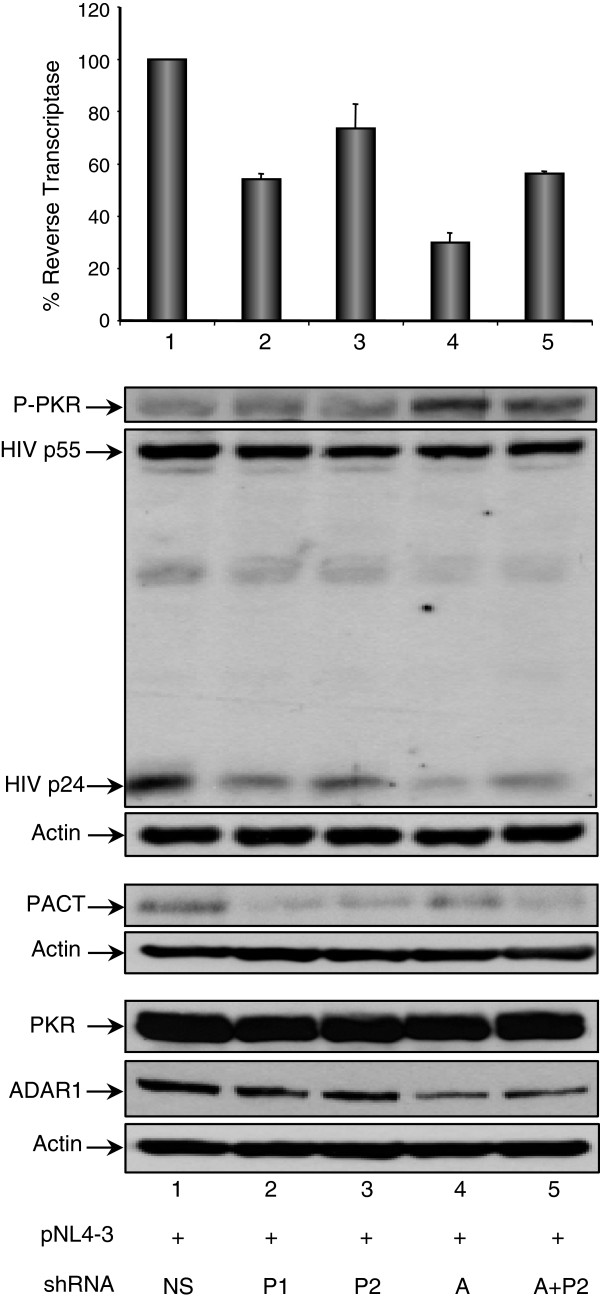
**shRNAs against PACT and ADAR1 decrease HIV-1 protein expression and virion production.** HEK 293T cells were co-transfected with 0.2 μg pNL4-3 (lane 1–5), and 2 μg of shRNAs NS (lane 1), 2 μg of shRNA1 PACT (lane 2), 2 μg of shRNA2 PACT (lane 3), 2 μg of shRNA ADAR1 (lane 4) and a combination of shRNA2 PACT and shRNA ADAR1 at 1 μg each (lane 5). 100 μg of cell lysates were resolved by SDS-PAGE and analyzed by immunoblotting using antibodies against P-PKR, PKR, HIV-1 p24, PACT, ADAR1, and actin. NS stands for non-specific, P1 and P2 stand for two different shRNAs against PACT and A stands for shRNA against ADAR1.

To further determine the role of endogenous PACT on HIV-1 expression and to compare with the function of ADAR1, we generated short hairpin RNAs (shRNAs) against PACT and against ADAR1 mRNAs to decrease their protein expression (Figure 5). Cotransfection of HEK 293T cells with pNL4-3 together with the shRNA1 or 2 against PACT (P1 and P2, two variants of the same sequence), the shRNA against ADAR1 or a combination of shRNA ADAR1 and shRNA P2 against PACT all decreased HIV-1 protein expression and viral production. Whereas both shRNAs PACT induced a modest increase of PKR phosphorylation, the shRNA ADAR1 consistently enhanced it. A combination of shRNA2 PACT and shRNA ADAR1 for the same final amount resulted in an intermediate effect on viral production compared to the two shRNAs alone (Figure 5, lane 5 compared to lanes 3 and 4), suggesting an additive effect. In agreement with the data with PACT overexpression, these results suggest that in these cells, PACT contributes to the enhanced HIV-1 protein production in combination with other proteins.

### PACT is a PKR inhibitor in HIV-1 transfected U251MG astrocytic cells

**Figure 6:**
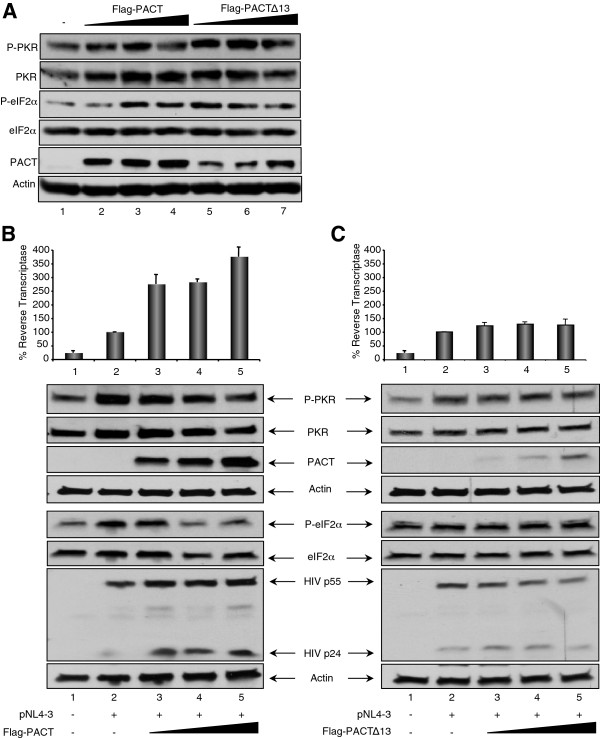
**PACT and PACT∆13 are not PKR activators in HIV-producing U251MG astrocytes. A)** PACT and PACT∆13 activate PKR and eIF2α in U251MG astrocytes. U251MG astrocytes were mock transfected (lane 1), transfected with 0.5 μg (lane 2 and 5), 1 μg (lane 3 and 6), 2 μg (lane 4 and 7) of pCMV2-Flag-PACT or pCMV2-Flag-PACT∆13 as indicated. 100 μg of cell lysates were resolved by SDS-PAGE and analyzed by immunoblotting using antibodies against P-PKR, PKR, HIV-1 p24, Flag and actin. **B)** PACT moderately inhibits PKR activation and causes an increase of HIV-1 production in U251MG astrocytes. U251MG astrocytes were mock transfected (lane 1), transfected with 2 μg pNL4-3 (lanes 2–5) and 0.5, 1 and 2 μg of pCMV2-Flag-PACT (lanes 3–5). The corresponding empty plasmid, pCMV2, was supplemented to each transfection to have the same amount of DNA transfected in each well. (Top) The RT activity is displayed as a percentage of activation compared to pNL4-3 alone. Results shown are an average of 3 independent transfections ± SEM. (Bottom) Cell extracts were immunoblotted and probed using antibodies against P-PKR, PKR, Flag, HIV-1 p24, P-eIF2α, eIF2α and actin. Shown is a representative experiment. **C)** PACT∆13 affects neither PKR activation nor HIV-1 production in U251MG astrocytes. U251MG astrocytes were mock transfected (lane 1), transfected with 2 μg pNL4-3 (lanes 2–5) and 0.5, 1 and 2 μg of pCMV2-Flag-PACT13 (lanes 3–5). The corresponding empty plasmid, pCMV2, was supplemented to each transfection to have the same amount of DNA transfected in each well. (Top) The RT activity was displayed as a percentage of activation compared to pNL4-3 alone. Results shown are an average of 3 independent transfections ± SEM. (Bottom) Cell extracts were immunoblotted and probed using antibodies against P-PKR, PKR, Flag, HIV-1 p24, P-eIF2α, eIF2α and actin. Shown is a representative experiment.

We next wanted to determine if the function of PACT as a PKR inhibitor during HIV-1 replication in lymphocytes as well as in HIV-1 production in HEK 293T cells could be due to TRBP heterodimers formation [18]. To do this, we evaluated PACT’s activity in U251MG astrocytic cells which naturally express low levels of TRBP. We first confirmed that Flag-PACT activates PKR in astrocytes and induces eIF2α phosphorylation as previously shown [18]. We also verified the activity of PACT∆13 (also called PACT305 or PACT∆1), a truncated PACT lacking 13 amino acids in its C-terminus that constitutively activates PKR and is poorly sensitive to TRBP inhibition. Similar to previous results [18], PACT and PACT∆13 induced PKR and eIF2α phosphorylation with PACT∆13 being highly active at low doses (Figure 6A). We then repeated the experiment in U251MG cells expressing HIV-1 proteins. Indeed, if PACT is a PKR activator in astrocytes expressing HIV-1 like in Figure 6A, it could be ascribed to the lack of TRBP-PACT heterodimer formation [18,20,55]. In contrast, if PACT is a PKR inhibitor in these cells, the effect would not solely be due to TRBP. We observed that overexpression of PACT in HIV-transfected U251MG astrocytoma cells induced an increased expression of HIV-1 protein production and inhibited PKR and eIF2α phosphorylation (Figure 6B). This result shows that PACT becomes a PKR inhibitor in astrocytes when these cells express HIV-1 proteins. The low level of HIV-1 virion production was increased by up to 4-fold, which is similar to what was previously observed with TRBP [12,49]. PACT∆13 is a potent PKR activator in HIV-non-infected astrocytes ([18] and Figure 6A) and mediates apoptosis through PKR activation in HT1080 cells [61]. In U251MG cells transfected with HIV-1, we observed that PACT∆13 lost its activating property on PKR and eIF2α phosphorylation (compare Figure 6C to 6A). Compared to wild-type PACT, it lost the enhancement in HIV-1 protein expression and virion production (compare Figure 6C to 6B).

### PACT and ADAR1 directly interact in cells

**Figure 7:**
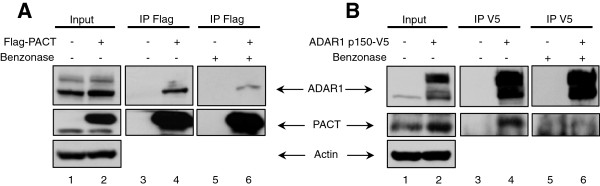
**PACT interacts with ADAR1. A)** Transfected Flag-PACT interacts with endogenous ADAR1. HEK 293T cells were not transfected (lanes 1, 3, 5), or transfected with 2 μg of pCMV2-Flag-PACT (lanes 2, 4, 6). IP was performed with 2 mg of protein and anti-Flag antibody and treated with Benzonase (lane 5, 6). 150 μg of cell lysate (input or immunoprecipitates) were resolved by 10% SDS-PAGE and analyzed by immunoblotting using antibodies against ADAR1, PACT and actin. **B)** Transfected ADAR1-V5 interacts with endogenous PACT. HEK 293T cells were not transfected (lanes 1, 3, 5), or transfected with 2 μg of pcDNA3.1-ADARp150-V5 (lanes 2, 4, 6). IP was performed with 2 mg of protein and anti-V5 antibody and treated with Benzonase (lane 5, 6). 150 μg of cell lysate (input or immunoprecipitates) were resolved by 10% SDS-PAGE and analyzed by immunoblotting using antibodies against ADAR1, PACT and actin.

The experiments in Figure 6 demonstrate that TRBP-PACT interaction cannot solely explain the change in PACT function in HIV-1 expressing cells. We therefore wanted to determine if this change could be due to a virally-induced mechanism. Because we showed that ADAR1 expression is induced during HIV-1 replication both in a lymphocytic cell line [12] and in PBMCs (Figure 1), we tested if ADAR1 and PACT could interact directly in cells. We transfected HEK 293T cells with either Flag-PACT or ADAR1 p150-V5, immunoprecipitated respectively with anti-Flag or anti-V5 antibody and blotted for either the endogenous ADAR1 or PACT (Figure 7). We recovered both proteins in the immunoprecipitate with either antibody, showing an interaction (Figure 7, lanes 4). When we treated the extracts with Benzonase, which contains RNAses against ss and dsRNAs, we clearly recovered ADAR1 with an anti-Flag showing an interaction between ADAR1 and PACT in the absence of RNA. The reverse IP with Benzonase was not as distinct, but also showed a likely direct interaction. Taken together, these results show that ADAR1 binds to PACT with or without RNAs, which could explain the reversal of PACT’s function in HIV-1 producing cells.

## Discussion

During HIV-1 infection, IFNα/β is mainly produced by plasmacytoid dendritic cells and acts on infected cells, but this cell response is not sufficient to clear the virus in patients [62]. Our results show that PBMCs do respond to IFN by producing IFN-stimulated genes (ISGs) and inhibiting HIV-1 replication (Figure 1). Therefore, the lack of *in vivo* efficacy cannot be ascribed to a lack of cell response to IFN. It could be due to either an insufficient amount of IFN production or to a block in the downstream effects of IFN or both. IFNα/β also has adverse effects, which limits its therapeutic use [63-65], emphasizing the need to better understand the downstream effects of ISGs and their regulation in HIV-1-infected cells. Among the ISGs, PKR and its activator PACT can either contribute to translational inhibition, proliferation arrest and apoptosis through eIF2α, I-κB phosphorylation or IFNβ induction when PKR is activated [52-54,61,66,67], or to increased viral replication and NF-κB signaling when it is not activated [12,17,25,26,68]. Because the PKR/PACT axis is part of the innate immune response to viruses, the elucidation of its activity is important to understand the inefficient response during HIV-1 replication. We and others have shown that PKR is extremely effective in restricting HIV-1 replication *in vitro*[12,27-30,49]. Furthermore, knocking down PKR by small interfering RNAs (siRNAs) or expressing a transdominant mutant of PKR increases HIV-1 production [49]. Despite this activity, HIV-1 replicates efficiently in many cells, suggesting that the activity of PKR in natural infection is highly regulated [17]. We therefore investigated the activation or deactivation of PKR during HIV-1 infection and the activity of exogenous IFN on PKR induction and activation. The transient activation of PKR followed by an absence of activation during HIV-1 infection of PBMCs (Figure 1) resembles the one observed with lymphocytic cell lines infected with X4 or R5 HIV-1 strains [12]. The transient activation of PKR in PBMCs suggests that this part of the innate immune response is active but is also tightly regulated during the infection of primary lymphocytes and monocytes in patients. Interestingly, the addition of IFN inhibited virus growth and induced PKR induction and activation. PKR induction was delayed by two days compared to the mock infection emphasizing that the presence of the virus postpones its expression. Furthermore, ADAR1 and PACT were induced at day 4 suggesting that an early protein from the virus may contribute to their expression.

The regulation of PKR activation is the result of the action of activators and inhibitors. The equilibrium reached after a viral infection contributes to a high or a weak cell response that will either activate innate immunity and block viral replication or let the virus replicate [32]. In the case of HIV-1 infection, the TAR RNA is likely one of the main activators of PKR at the beginning of the infection, but may become an inhibitor if produced in large amounts in the cell [69]. The HIV-1 Tat protein is also an inhibitor of PKR acting by substrate competition [31]. Besides direct viral countermeasures, viruses also evolved to replicate in cells that have the appropriate cellular components to allow their replication [70]. Viruses can also induce the production of cellular proteins that will counteract an antiviral cell response. A cell that expresses high amounts of PKR inhibitors certainly favors HIV-1 replication. HIV-1 replicates in cells that express a large amount of TRBP that inhibits PKR [39,49]. HIV-1 also induces ADAR1 production, which contributes to PKR inhibition and RNA editing and favors viral replication [12,50,71]. We show here that ADAR1 is also induced in PBMCs (Figure 1B), which corroborates this effect in primary cells. Because TRBP not only acts on PKR, but also prevents PACT activity on PKR [18,55], we originally thought that PACT may activate PKR and that the end-up result of the PKR status would be a balance between PKR activators and PKR inhibitors. The identification of PACT in a protein complex with PKR, TRBP and ADAR1 during HIV-1 infection suggested a role for PACT but raised the question of its function within this complex (Figure 2).

When overexpressed in HIV-1-expressing cells, PACT inhibited PKR and eIF2α phosphorylation and consequently increased HIV-1 expression (Figures 3 and 4). PACT inhibition of PKR activation and consequently on eIF2α phosphorylation was very dramatic on endogenous PKR (Figures 3A and 4A), indicating that PACT reverses its function in HIV-1-producing cells. When PKR was overexpressed, the effect of PACT on PKR activation was only visible on the P-PKR/PKR and P- eIF2α/eIF2α ratio (Figure 3B), but clearly reversed PKR inhibition of HIV-1 production, suggesting that PACT may also act through another kinase like PERK, or directly on eIF2α, or it may have an additional activity to increase viral expression. The mechanism of this increased viral expression despite high PKR phosphorylation may be related to the phosphorylation of HIV-1 Tat by PKR [72] or to a transcriptional activity of PACT similar to the recently observed recruitment of PACT, TRBP and Dicer to the promoter of nuclear receptors [73]. Furthermore, PACT inhibition by shRNAs decreased HIV-1 protein expression similarly to shRNAs against ADAR1 (Figure 5). Together, the increased expression of PACT during HIV-1 replication in PBMCs (Figure 1), the increased PKR-PACT interaction at the peak of infection (Figure 2), PACT activity upon over-expression (Figures 3 and 4) and results with shRNAs (Figure 5) contribute to reach the same conclusion that PACT is a PKR inhibitor during HIV-1 replication. There are several explanations that could explain this activity: i) TRBP is present in high amounts in HEK 293 T cells and forms heterodimers with all PACT molecules, which reverses PKR activation; ii) the large amount of ADAR1 induced by HIV-1 binds to PACT and reverses its function; or iii) an HIV-1 component or an HIV-induced component will change PACT from an activator into an inhibitor of PKR.

Our results in the astrocytic cells U251MG show that the first hypothesis by the formation of TRBP-PACT heterodimers cannot be the sole explanation, strongly suggesting that an HIV-1 component or an HIV-induced component mediates the change in PACT function in HIV-1-expressing cells (Figure 6). This component prevents PACT from being a PKR activator and changes it into a PKR inhibitor with a similar activity as TRBP on PKR. Furthermore, PACT ∆13, although not naturally produced in cells, has been shown to be a strong activator of PKR, because it is constitutively active and not regulated by TRBP [18,20,61]. Its loss of activity in HIV-1-expressing astrocytes reinforces the idea that an HIV-1 or HIV-induced component reverses PACT activating function on PKR independently of TRBP (Figure 6C). The second possibility would be that the HIV-mediated increase in ADAR1 expression mediates a change in PACT function by direct binding. Our IP assays show that it may be the case because the two proteins are in the same complex during HIV-1 infection (Figure 2) and that they interact in the absence of RNA (Figure 7). We cannot exclude that another mechanism may be involved as well. PACT-Dicer interaction [74] or PACT induction of RIG-I upon Sendai virus infection [75] seems unlikely here because it would lead to viral restriction or enhanced innate immune response respectively, which we do not observe during HIV-1 infection or after PACT overexpression of HIV-1-expressing cells (Figures 1, 3, 4, 6). Therefore, ADAR1-PACT interaction is currently the most likely mechanism, which may contribute, at least in part, to the change in PACT activity during HIV-1 infection.

Our results show that three cellular proteins, TRBP, ADAR1 and PACT contribute to the inhibition of PKR and eIF2α phosphorylation observed in HIV-1-infected cells (Figure 4). All of them are dsRBPs, therefore raising the question if all proteins of this family act similarly. Staufen1 was used as another dsRBP that has a positive activity on the virus by binding to Gag and by increasing translation from TAR-containing RNAs [9,76]. In agreement with its PKR-independent mechanism on translation [9], we found that Staufen1 did not inhibit PKR activation supporting a combined inhibition of PKR by TRBP, ADAR1 and PACT and a different mechanism for Staufen1 via Gag multimerization, HIV-1 assembly and encapsidation of genomic RNA [77-79], all contributing to viral replication. Further studies will determine if PKR forms a different protein complex at the beginning of HIV-1 infection when PKR and eIF2α are activated, if PACT has a different activity in this context and how it may contribute to the pathogenicity induced by the virus.

## Conclusions

Previous results have characterized PACT as a stress-inducible PKR activator. In contrast, we show here that PACT becomes a PKR inhibitor during HIV-1 replication. PACT belongs to a multiprotein complex including the PKR inhibitors TRBP and ADAR1 formed around PKR during high viral replication. Results strongly suggest that PACT reversion of PKR activation comes in addition to its control by TRBP and could be due to its interaction with ADAR1 and other HIV-1 or an HIV-induced component. These data show that HIV-1 has evolved using several mechanisms to overcome the innate cell response.

## Methods

### Cells and transfections

HEK 293T (ATCC CRL-11268) and U251MG [49] cells were maintained at 37°C in 5% CO_2_ in Dulbecco’s modified Eagle’s Medium (DMEM; Invitrogen) supplemented with 10% fetal bovine serum (HyClone), 2 mM L-glutamine, and 1% penicillin-streptomycin (Invitrogen). Jurkat T cells (ATCC TIB-152) were maintained in RPMI-1640 (Invitrogen) supplemented similarly.

Peripheral blood mononuclear cells (PBMCs) were obtained from healthy donors previously selected to be negative for HIV, HTLV-I and II, HCV, CMV and syphilis. Blood sample collection was approved by the ethics review board of McGill University.

For transfection of HEK 293T and U251MG cells with plasmids, cells were plated in six-well plates at 50% confluence 24 h prior to transfection using polyethylenimine (PEI) following manufacturer’s protocol (Polysciences). Transfection of HEK 293T cells with shRNA vectors (2 μg/well in a 6-well plate) was performed 24 h after plating using TransIT-LT (Mirus) as described [80]. pNL4-3 (0.2 μg/well in a 6-well plate) was then transfected using TransIT-LT 24 h after transfection of the shRNAs. Supernatants and lysates were then collected 48 h after transfection of pNL4-3 as described [81].

### Plasmids and shRNA synthesis

HIV-1 clone pNL4-3, pcDNA1-PKR, pCMV2-Flag-PACT, pCMV2-Flag-PACT∆13, pcDNA3-TRBP2, pcDNA3.1-ADARp150-V5, and pcDNA3-RSV-Staufen1-HA were previously described [12,55,82].

shRNAs targeting PACT and ADAR1 were cloned into the psiRNA vector (InvivoGen) using sequences obtained from the Sigma-Aldrich website. The sense (S) and antisense (AS) oligonucleotide sequences of the shRNAs are:

PACT 1: (S) 5′ - ACCTCGCGCCAATGGACAATATCAATCTCGAGATTGATATTGTCCATT GGCGCTT - 3′ and (AS) 5′ - CAAAAAGCGCCAATGGACAATATCAATCTCGAGATTGATATTGTCC ATTGGCGCG - 3′. PACT 2: (S) 5′ - ACCTCGCGCCAATGGACAATATCAATACTCGAGAATTGATATTGTCCATT

GGCGCTT- 3′ and (AS) 5′ -

CAAAAAGCGCCAATGGACAATATCAATTCTCGAGTATTGATATTGTCCATTGGCGCG - 3′.

ADAR1:(S) 5′ - ACCTCGCTGTTAGAATATGCCCAGTTACTCGAGAAACTGGGCATATTCTA

ACAGCTT- 3′ and (AS) 5′ -CAAAAAGCTGTTAGAATATGCCCAGTTTCTCGAGTAACTGGGCATATTCTAACAGCG - 3′.

After annealing at 80°C for 2 minutes, the shRNAs were ligated into BbsI-digested psiRNA.

### Transfection of HIV-1 molecular clones and RT assay

HEK 293T were transfected as above with pNL4-3 proviral DNA. Cell supernatants were collected 48 h post transfection and viral production assayed for standard RT assay. RT assay was as previously described [81] with modifications described in [83]. Supernatants from transfected HEK 293T cells were used for infection of Jurkat or PBMCs.

The RT assay from the supernatant of transfected U251MG cells was carried out in a similar manner, with the exception of a longer incubation period at 37°C (3 h) and 10 μl being spotted onto the DEAE paper as previously [84]. This was to account for the low amount of virus production in U251MG astrocytes.

### HIV-1 viral infection of Jurkat cells and PBMCs

HIV-1 Jurkat cells infection was previously described [12]. For PBMCs HIV-1 infection, cells were stimulated with 0.6 μg/ml phytohaemagglutinin (Sigma cat. # 12646) for three days in supplemented RPMI (Invitrogen). 24 h prior to infection, recombinant human interleukin 2 (IL-2) (R&D Systems, cat. # 202-IL) was added to the cells for a final concentration of 10 ng/ml. 6.5 × 10^7^ cells were infected with HIV-1 cell supernatant corresponding to 1.3 × 10^7^ cpm measured by standard RT assay in a final volume of 2.5 ml supplemented RPMI in polypropylene round-bottom tube, and incubated for 2 h at 37°C. RPMI supplemented with IL-2 for a final concentration of 10 ng/ml was then added to the cell-virus mixture, transferred to a T25 flask and incubated at 37°C. The cells were fed on average every two days with fresh medium supplemented with IL-2 (10 ng/ml). Supernatant and cell samples were collected at different times and assayed for RT activity, immunoblotting and IP when indicated.

### Immunoprecipitation and immunoblotting

IP with infected Jurkat cells was previously described [12]. For IP from HEK 293T cells, 48 h post-transfection, cells were washed twice with PBS and lysed in the cold lysis buffer with protease inhibitors. For each IP, 50 μl of protein G agarose fast flow compact beads (Sigma) were washed with TNEN (50 mM Tris–HCl [pH 7.4], 100 mM NaCl, 1 mM EDTA [pH 8], 0.5% NP40 (Sigma)) and left rotating at 4°C for overnight incubation at 4°C with 5 μg of anti-V5 antibody (Invitrogen). 500 μg to 2 mg of cell extract was added to the beads for overnight incubation at 4°C. The beads were washed 3 times with 1 ml of cold lysis buffer, 5 times with 1 ml cold PBS and resuspended in SDS loading dye. When indicated, the beads were treated with 250 U/ml of Benzonase® (Sigma) in 50 mM Tris–HCl, 1 mM MgCl2, pH 8.0 for 30 min at 37°C. Bound proteins were eluted by boiling the beads for 5 min and separated by 10% SDS-PAGE. The immunoprecipitates were analyzed by a Western blot analysis using the anti-ADAR1 (from Dr. BL Bass) or anti-PACT (Medimabs) antibodies.

For immunoblotting, HEK 293T, or Jurkat T cells extracts were prepared, separated and transferred on a Hybond ECL nitrocellulose membrane (GE Healthcare) as previously described [55]. Membranes were blocked for 1 h in 5% nonfat dry milk and Tris-buffered saline-0.1% Tween 20 (TBST). Membranes were incubated overnight at 4°C with the primary antibody. After five washes in TBST, membranes were incubated with Horseradish Peroxidase-conjugated secondary goat anti-rabbit or goat anti-mouse antibody (GE Healthcare). Anti-P-PKR (Abcam) and anti-P-eIF2α (Invitrogen) was blotted in 3% BSA/TBST overnight. After immunoblotting with an antibody, the membranes were washed in TBST overnight or stripped and reused to detect other proteins. The bands were visualized using ECL (GE Healthcare). Primary antibodies used for immunoblotting were: monoclonals anti-PKR 71–10 [85] obtained from Dr. A. Hovanessian, anti-Actin (Chemicon) at a 1/5000 dilution, anti-HIVp24 183-H12-5C [86], at a 1/1000 dilution, anti-Flag (Sigma) at a 1/5000 dilution, polyclonal anti-TRBPjbx [18] at a 1/500 dilution, anti-P-PKR (Abcam), anti-PACT (Medimabs), anti-Staufen1, anti-ADAR1, anti-P-eIF2α (Invitrogen) and anti-eIF2α (Cell Signaling), at a 1/1000 dilution. The anti-Staufen1 antibody was generated at the Cell Imaging and Analysis Network (McGill University, Montréal, Canada) using purified full-length His-tagged Staufen1 protein. For multiple blotting, when the same membrane cannot be blotted again, extracts from the same experiment were separated by SDS PAGE and blotted on a new membrane. In this case, actin is shown for each membrane. Where indicated, the bands where quantified by densitometry analysis as described [80].

## Abbreviations

HIV-1: Human immunodeficiency virus type 1; PKR: Protein kinase RNA-activated; eIF2α: Alpha subunit of the eukaryotic translation initiation factor 2; IFN: Interferon; dsRBP: Double-stranded RNA binding protein; dsRBD: Double-stranded RNA binding domain; ADAR1: Adenosine deaminase acting on RNA 1; TRBP: TAR RNA binding protein; PACT: PKR activator; PBMCs: Peripheral blood mononuclear cells; siRNA: Small interfering RNA; shRNA: Short hairpin RNA; RT: Reverse transcriptase; IP: Immunoprecipitation.

## Competing interests

The authors declare that they have no competing interests.

## Authors’ contributions

The work was originally designed by GC, JFG and AG, followed by ES, AD, SB, TE, LS and AG. GC, ES, AD, SB, JFG, TE and LS performed the experiments and analyzed the corresponding data. JPR, AJM, RCP provided reagents and analyzed data. AG has supervised all the work and wrote the final version. All authors have participated to the writing and have approved the final version.
